# Effect of Ripening on the Phenolic Composition and Mineral Content of Three Varieties of Olive Fruits

**DOI:** 10.3390/foods10020380

**Published:** 2021-02-09

**Authors:** María del Pilar Fernández-Poyatos, Eulogio J. Llorent-Martínez, Antonio Ruiz-Medina

**Affiliations:** Department of Physical and Analytical Chemistry, Faculty of Experimental Sciences, University of Jaén, Campus Las Lagunillas, E-23071 Jaén, Spain; mpoyatos@ujaen.es (M.d.P.F.-P.); ellorent@ujaen.es (E.J.L.-M.)

**Keywords:** olives, *Olea europaea*, Cornezuelo, Cornicabra, Picual, phenolic, oleuropein

## Abstract

The phenolic composition and mineral content of Cornezuelo, Cornicabra and Picual olive fruit varieties were investigated during olive ripening in two different harvesting seasons (2017/2018 and 2018/2019). Phytochemical profiles were evaluated by high-performance liquid chromatography (HPLC) with diode-array and mass spectrometry detection. Mineral contents were determined by inductively coupled plasma-mass spectrometry (ICP-MS). Twenty-five compounds were characterized and the main ones quantified. These compounds corresponded mostly to secoiridoids, the main ones being oleuropein, oleoside/secologanoside, oleoside-11-methylester, and oleuropein and comselogoside isomers. Total phenolic contents reached the highest values between December and January, coinciding with the usual harvesting date. This trend was observed in both harvesting seasons, although higher phenolic contents were recorded in season 2018/2019. This was due to the different weather conditions, which caused a lower olive production in season 2017/2018. No clear tendency was observed between mineral content and harvest time in any of the studied seasons. The highest concentration of total phenolics was obtained in Cornezuelo variety (840 mg/100 g) in January 2019 (season 2018/2019). Picual and Cornicabra varieties reached concentrations of 670 mg/100 g and 530 mg/100 g, respectively, also in the last harvesting dates of season 2018/2019.

## 1. Introduction

*Olea europaea* L., belonging to the Oleaceae family, is one of the most important fruit trees in the Mediterranean area. It is widely cultivated in countries of southern Europe that border the Mediterranean Sea and in North Africa [1]. Spain has more than 2 million and a half hectares of olive groves, being the main producer of fresh olives, ahead of Italy and Greece, and the first producer in the world for table olives [2]. The quality of the olive fruits is affected by maturity at the time of harvest [3]. The phenolic composition—which is important in several aspects—varies qualitatively and quantitatively during fruit ripening [4]. These biochemical changes affect the texture, flavour and colour, which determine the organoleptic and nutritional quality of the fruits [5]. The main classes of phenols in olive fruits are phenolic acids, phenolic alcohols, flavonoids, and secoiridoids, with the last one being exclusively present in the Oleaceae family [6]. Oleuropein is the principal secoiridoid present in olive fruits. The great interest in olive polyphenols is due to their importance for human health, being a very healthy complement in the Mediterranean diet. In fact, numerous studies have shown evidence for their therapeutic and nutritional properties [7]. For instance, the decline of people with cardiovascular diseases in the Mediterranean Basin has been attributed in part to the consumption of olive products [8]. Polyphenols act as antioxidants and radical scavengers and are considered safe sources of natural antioxidants in the food industry [9]. 

Two of the most typical olive varieties in Spain are Cornicabra and Picual, known for the excellent quality of their (extra) virgin olive oils. Several studies have been carried out in these two varieties to study the effect of olive ripening on the content of polyphenols in olive fruits [10,11,12,13] and olive oils [10,14,15]. On the other hand, the Cornezuelo variety is less known, and the few studies regarding this variety have focused on olive oil, including organoleptic characteristics, fatty acids, triglycerides, and sterols [16,17]. However, Cornezuelo olives are increasing their popularity due to their excellent organoleptic characteristics, so the composition of their fruits was recently reported [18]. To our knowledge, this is the first report on the effects of the ripening process on Cornezuelo olive fruits.

The main purpose of this work was to identify and quantify phenolic compounds during the maturation process of the fruits of three Spanish cultivars (Cornezuelo, Cornicabra and Picual) in two different harvesting seasons and compare the results among the varieties. The secondary objective was to compare the concentration of phenolics in Cornezuelo (the least studied variety among the three here discussed) with Cornicabra and Picual. Additionally, the inorganic content of each of the varieties during their maturation was measured by inductively coupled plasma–mass spectrometry (ICP-MS), not only to provide data concerning their mineral content but also to ensure the absence of heavy metals considering that these olive fruits are intended for the production of olive oil. The main hypothesis is that phenolic content will vary between the different olive varieties and during the maturation process (following a similar trend concerning variations in concentration). The second hypothesis is that phenolic levels will be higher on the usual collection dates, when the maturation of the olives is considered optimal. The last hypothesis is that the phenolic content of Cornezuelo olive fruits will be similar or higher than the contents of the other two varieties.

## 2. Material and Methods

### 2.1. Chemicals and Reagents

All analytical standards were purchased from Sigma-Aldrich (Madrid, Spain). Solutions were prepared in HPLC-grade methanol (Sigma-Aldrich). LC-MS grade formic acid (HCOOH), acetonitrile (CH_3_CN) and ultrapure water were also used. 

Hydrogen peroxide (H_2_O_2_; ultra-trace analysis) and nitric acid (HNO_3_; 65%; purified by sub-boiling distillation) were purchased from Panreac (Madrid, Spain). HPLC-grade n-hexane (>95%) was obtained from Alfa Aesar (Haverhill, MA, USA).

For ICP-MS analysis, NIST-3281 (cranberry fruit) certified reference material was used. 

### 2.2. Sample Preparation and Extraction

Olives cv. Cornicabra and cv. Picual were collected by hand in an olive grove in Albanchez de Mágina (Jaén, southeast of Spain; 37°47′40.6″ N 3°28′17.3″ W, 891 m a.s.l.) and Cornezuelo olive fruits were picked up in Torres (Jaén, southeast of Spain; 37°46′58.8″ N 3°30′59.9″ W, 894 m a.s.l.).

The study was performed during two consecutive harvesting seasons. The first season corresponded to the olive fruits collected in 2017/2018 on three different harvest dates (HD): 1 November 2017 (2nd HD); 6 December 2017 (3rd HD); 10 January 2018 (4th HD). During this season, samples were not collected before November because olive fruits were still scarce and very small. The second season corresponded to the samples collected in 2018/2019 on four dates: 19 October 2018 (1st HD); 16 November 2018 (2nd HD); 13 December 2018 (3rd HD); 16 January 2019 (4th HD). The last HD for both seasons corresponded to the final HD performed by olive farmers. Compounds identification for each HD is shown in Table 1.

Maturity indexes (MI) for each variety during the different HDs are shown in Table 2, Table 3 and Table 4. MI was determined on 100 randomly selected olives [19]. 

Phenolic compounds were extracted using a previous procedure [20] with modifications. An amount of 2 g of olives (pitted and crushed) was extracted with 30 mL of MeOH:H_2_O (80:20, *v*/*v*) for 10 min using an ultrasonic liquid processor at 50% power (Qsonica Sonicators; Newton, CT, USA; 55 W power and 20 kHz frequency). After centrifugation at 1.800 g for 15 min, solutions were filtered (Whatman No.1 filters). Solutions were subjected to clean-up with 40 mL of n-hexane (twice). The solvent was evaporated under reduced pressure and dried extracts (DE) were stored at −20 °C. Extractions were performed in triplicate for each sample.

### 2.3. HPLC Analysis of the Phenolic Compounds

All chromatographic conditions are given in Supplementary Material.

### 2.4. ICP-MS Analysis of Olives

The characteristics of the equipment, operating conditions and validation of ICP-MS method are included in Supplementary Material (Tables S1–S3 and Figure S1).

Sample digestion was performed with a MARSXpress microwave digestion system (CEM; Gilson; Madrid, Spain) with 50 mL PFA vessels. The following procedure was used (in triplicate for each sample): 0.25 g of sample (pitted and homogenized), 7 mL HNO_3_ and 3 mL H_2_O_2_ were added to a digestion vessel. After 10 min at room temperature, samples were digested as detailed in Supplementary Material in Table S4. Then, the solutions were diluted to 50 mL with ultrapure water in metal-free ICP-MS vials.

Calibration curves were prepared at six concentrations in 5% (*v*/*v*) sub-boiling HNO_3_. The ICP-MS instrument added internal standards automatically.

### 2.5. Statistical Analysis

SPSS Statistics software v.22 (IBM SPSS Statistics for Windows, IBM Corp., Armonk, NY, USA) was used. Data are expressed as mean ± standard deviation (three replicates). A one-way analysis of variance (ANOVA) with Tukey’s HSD post-hoc test (*p* < 0.05) was done.

## 3. Results and Discussion

### 3.1. HPLC-MS Analysis

Initially, for the 2017/2018 season, the months of November, December and January were selected to analyse the olive fruits, as they are the typical months during which farmers usually harvest them. The phenolic contents obtained in this harvest were low compared to previously reported ones [10]. This could be due to the low olive production and low fat content in the 2017/2018 harvest, probably caused by the weather conditions (rain precipitations in the months previous to the 2017/2018 harvesting were approximately 50% of those in the following season). Therefore, two objectives were proposed to improve this study: to repeat the analyses for season 2018/2019; and to analyse olive fruits also in October (due to the recommendation of several experts regarding early harvesting of fruits to improve olive oil characteristics). 

**Figure 1:**
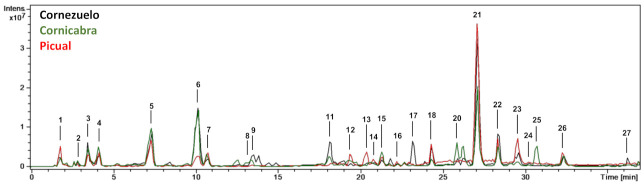
HPLC-ESI/MS^n^ base peak chromatograms (BPC) of the extracts of Cornezuelo, Cornicabra and Picual olives for 2018/2019 season in 4th HD.

**Figure 2:**
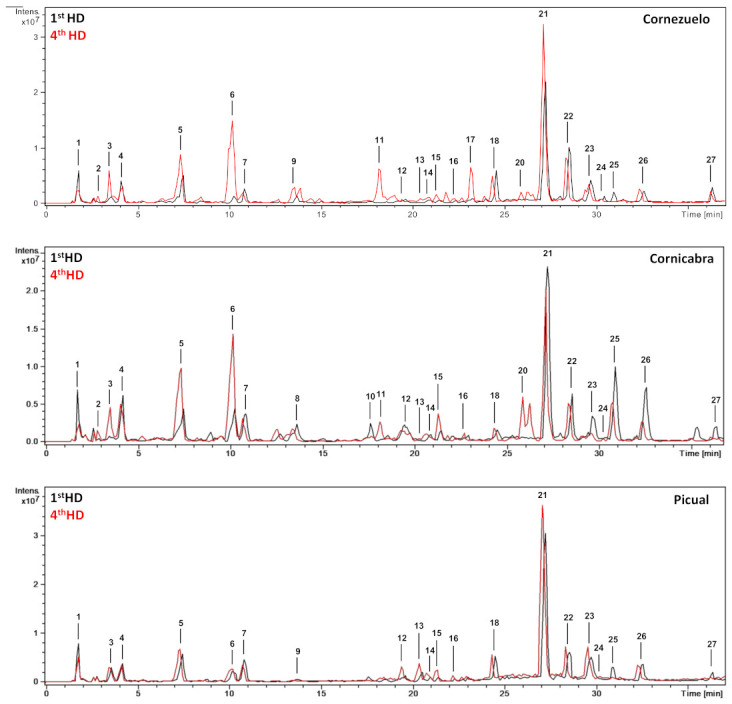
HPLC-ESI/MS^n^ base peak chromatograms (BPC) of the extracts of Cornezuelo, Cornicabra and Picual olives in 1st HD and 4th HD (2018/2019 season).

From a qualitative point of view, the base peak chromatograms of the three olive fruit varieties (season 2018/2019, 4th HD) showed similar profiles (Figure 1). Chromatograms comparing the 1st HD and 4th HD (October and January, respectively) of each olive variety (Cornezuelo, Cornicabra and Picual) for the 2018/2019 season are shown in Figure 2. A total of 25 phytochemicals, almost 70% secoiridoids, were identified or putatively characterized. The characterization was done by using analytical standards and bibliographic information. Compounds were numbered according to their order of elution, keeping the same numeration in all samples. Mass spectrometry data are given in Table 1 for the compounds identified in season 2018/2019 (similar results were obtained in season 2017/2018 for the same HD).

#### 3.1.1. Secoiridoids

Compound 4 showed [M-H]^−^ at *m/z* 407 and its fragmentation pattern was in agreement with 1-β-glucosyl-acyclodihydroelenolic acid [21]. 

Compounds 5, 6, 18, and 27 were oleoside derivatives. Compound 5 presented [M-H]^−^ ion at *m/z* 389 and suffered the loss of 44 Da (carbon dioxide molecule) to yield the base peak at *m/z* 345; it was tentatively characterized as oleoside/secologanoside [22]. Compound 6 was characterized as oleoside-11-methylester with an analytical standard. Compound 18 was another oleoside, 6′-β-hexopyranosyloleoside, based on its [M-H]^−^ ion at *m/z* 551, MS^2^ base peak at *m/z* 507, and MS^3^ base peak at *m/z* 161 [23]. According to bibliographic data, compound 27, with deprotonated molecular ion at *m/z* 557, MS^2^ base peak at *m/z* 513, and MS^3^ at *m/z* 345, was tentatively characterized as an oleoside/secologanoside derivative [23]. 

Compounds 7, 10, 15, 16, 17, 19, 21 and 23 were oleuropein derivatives. Compounds 7 and 19, with [M-H]^−^ at *m/z* 377, were identified as oleuropein aglycone isomers [24,25]. Based on the presence of a deprotonated molecular ion at *m/z* 525 and fragment ions at *m/z* 481, 389, 209 and 195, compound 10 was tentatively characterized as dimethyl-oleuropein glucoside [26]. Compound 15 was identified as dihydrooleuropein; it showed [M-H]^−^ at *m/z* 543 and main fragment ions at *m/z* 525 and *m/z* 513 [27]. With [M-H]^−^ at *m/z* 701, compounds 16 and 17 were identified as oleuropein glucoside isomers. Their MS^2^ fragmentation profiles produced main fragment ions at *m/z* 539, due to the neutral loss of a hexose (162 Da), and the typical fragmentation of oleuropein (*m/z* at 377, 307, and 275) was observed [22]. Oleuropein (compound 21) was one of the most abundant compounds and was identified by comparison with an analytical standard, observing characteristic fragments at *m/z* 377, 307 and 275 [25]. Compound 23 presented the same [M-H]^−^ and similar fragmentation pattern than oleuropein, so it was tentatively characterized as an isomer.

Compound 9 exhibited [M-H]^−^ at *m/z* 403 and was characterized as elenolic acid glucoside based on the fragment ions at *m/z* 371, 223 and 179, corresponding to neutral losses of 32 Da (methyl group), 180 Da (hexose) and 44 Da (carbon dioxide), respectively [28]. Compound 20 presented a fragmentation pattern similar to compound 9, so it was characterized as an elenolic acid derivative.

Compounds 22 and 24 exhibited deprotonated molecular ions at *m/z* 535, base peak at *m/z* 491, and fragmentation patterns typical of comselogoside [29]. 

Compound 26 was characterized as ligstroside due to the [M-H]^−^ at *m/z* 523, neutral loss of 162 Da (hexoside) to yield the base peak at *m/z* 361, and main fragment ions in MS^3^ at *m/z* 291 and 259 [22]. 

#### 3.1.2. Other Compounds

Compound 1, with deprotonated molecular ion at *m/z* 191 and characteristic fragment ions at *m/z* 173 and 111, was identified as citric acid [30]. 

Compound 2, with [M-H]^−^ at *m/z* 375, was characterized as dihydrocominic acid considering bibliographic information [31]. 

Compound 3 was identified as hydroxytyrosol glucoside. It presented [M-H]^−^ ion at *m/z* 315, fragment ion at *m/z* 153 (from the neutral loss of 162 Da, hexoside) and a typical fragment ion at *m/z* 123 [32]. With [M-H]^−^ ion at *m/z* 701 and the same fragmentation pattern that hydroxytyrosol glucoside, compound 11 was tentatively characterized as a derivative.

Compound 12, with [M-H]^−^ at *m/z* 609, was identified as rutin by comparison with an analytical standard.

Compound 13 was identified as verbascoside. It showed [M-H]^−^ ion at *m/z* 623 and characteristic fragments at *m/z* 461 (neutral loss of 162 Da, hexoside), *m/z* 315 (neutral loss of 146 Da, rhamnoside) and *m/z* 135. The identification was confirmed with an analytical standard.

Compound 14 was identified as luteolin-*O*-hexoside due to the neutral loss of 162 Da to yield luteolin aglycone at *m/z* 285.

### 3.2. Quantification of Phenolic Compounds

Twenty-one different compounds were quantified. The concentrations obtained for the three varieties of olive fruits, in the two different seasons, are given in Table 2, Table 3 and Table 4. 

A similar pattern with regards to the main compounds, as well as the trend observed during the different harvesting dates, was observed in both harvesting seasons. However, the three fruit varieties presented higher concentration of phenolics in season 2018/2019. The lower phenolic content in season 2017/2018 may be due to two different issues: low fruit production and olive yield, along with a higher percentage of humidity, which has been previously related to lower phenolic concentrations [15]. All humidity percentages are shown in Table S5.

First of all, it can be observed that the Cornezuelo variety presented the highest TIPC (Total Individual Phenolic Content: sum of all the quantified phenolics), followed by the Picual and Cornicabra varieties. Secoiridoids accounted for approximately 90% of TIPC in the Cornezuelo and Cornicabra varieties in both harvesting seasons. In the Picual variety, secoiridoids accounted for 70–90% of TIPC, with differences depending on the harvest dates; the highest percentage was obtained in 2018/2019 crop season.

**Figure 3:**
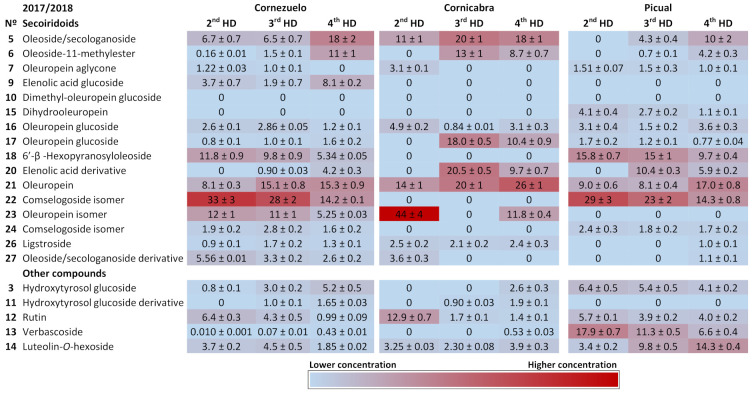
Heatmap showing the quantification of compounds in extracts of Cornezuelo, Cornicabra and Picual olives during the season 2017/2018 in different ripening stages using a colour scale. Data are given as percentage ± standard deviation (*n* = 3).

**Figure 4:**
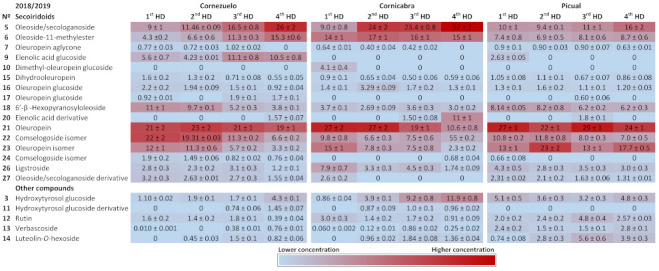
Heatmap showing the quantification of compounds in extracts of Cornezuelo, Cornicabra and Picual olives during the season 2018/2019 in different ripening stages using a colour scale. Data are given as percentage ± standard deviation (*n* = 3).

Figure 3 and Figure 4 show heat maps highlighting the main compounds in each of the olive varieties during both harvesting seasons. The main secoiridoids in all samples were oleuropein and its isomer, oleoside/secologanoside, oleoside-11-methylester and comselogoside isomers. Oleuropein and its isomer (compounds 21 and 23) accounted for 20–40%, 20–50%, and 10–50% of total compounds in the Cornezuelo, Cornicabra, and Picual varieties, respectively. A comselogoside isomer (compound 22) was most abundant in the Cornezuelo and Picual varieties (approximately 10–30%) compared to Cornicabra (lower than 10% of total compounds). On the other hand, oleoside/secologanoside (compound 5) was more abundant in Cornicabra (10–30%) than in Cornezuelo and Picual (Picual presented the lower percentage of this compound). In the Cornezuelo variety, the main secoiridoids mentioned represented approximately 70% of the total secoiridoids for both seasons, whereas in Cornicabra olive fruits they accounted for 80% of the total secoiridoids. In the Picual variety, the main secoiridoids showed wider variations (60–80% of the total secoiridoids) than those observed in the other olive varieties studied.

Regarding the content of individual phenolics, the maximum values of oleuropein in seasons 2017/2018 and 2018/2019, respectively, were 88 and 160 mg/100 g fresh weight (FW) in Cornezuelo, 80 and 72 mg/100 g FW in Cornicabra, and 44 and 191 mg/100 g FW in Picual. In previous studies in seven Spanish olive varieties, including Cornicabra and Picual, maximum oleuropein levels ranged between 178 mg/100 g and 1861 mg/100 g FW [10,13,33]. In other studies carried out in varieties from other countries, oleuropein levels similar to those obtained in this research were reported, such as 124–327 mg/100 g FW in the Portuguese Cobrançosa variety [34], 266–675 mg/100 g FW in the Tunisian olive varieties Dhokar, Chétoi and Chemlali [35,36] and 298 mg/100 g FW in the Turkish variety Sariulak [2]. These differences can be attributed not only to studies performed in other varieties, but also to changes in collection areas and climatological conditions. As can be observed by the results reported here, the specific season of olive harvesting is critical with respect to phenolic concentrations. Hence, a straightforward comparison of particular phenolic compounds is difficult to do between different studies.

For rutin, the maximum values obtained in our study ranged between 8.7 mg/100 g FW in Cornicabra and 32 mg/100 g FW in Picual. In a previous report, similar rutin values were observed: 24 mg/100 g FW in the Picolimón variety and 45 mg/100 g FW in Picual [10]. Similar values (17–54 mg/100 g FW) were reported in the Spanish varieties Arbequina and Royal [13,33], as well as in the Turkish varieties Sariulak, Ayvalik and Gemlik [2,37]. 

For verbascoside, the variety with the lowest concentration was Cornicabra (1.54 and 3.08 mg/100 g FW in seasons 2017/2018 and 2018/2019, respectively); these values are very similar to that previously reported in literature in Cornicabra olives (3.5 mg/100 g FW) [10]. The highest concentration of verbascoside was observed in the Picual variety (26 mg/100 g FW), in agreement with a previous study that also reported that the Picual variety had a high concentration of this compound (124 mg/100 g FW) [13]. In other varieties, concentrations of 36 mg/100 g FW in the Spanish variety Royal [33] and 111 mg/100 g FW in the Turkish variety Sariulak [2] were reported.

**Figure 5:**
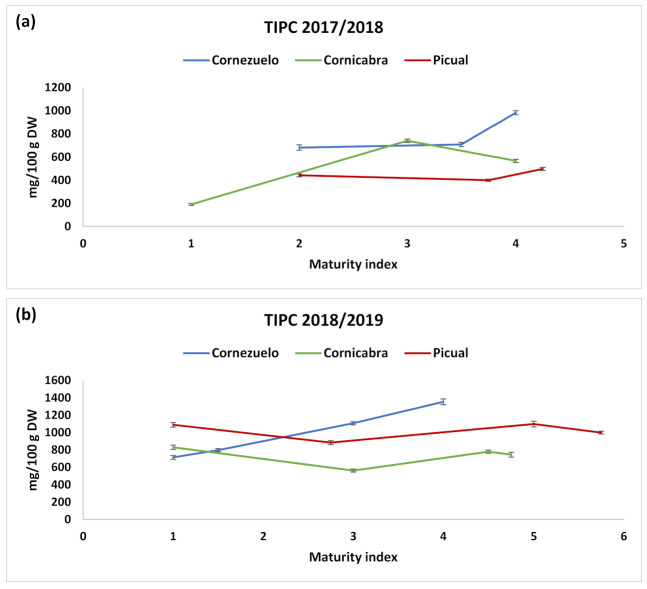
TIPC of the three varieties of olive fruits in the different collection months during seasons 2017/2018 (**a**) and 2018/2019 (**b**). Values (mg/100 g dry weight) are mean ± SD of three parallel measurements.

Comparing Cornezuelo with Cornicabra and Picual, it could be observed that the TIPC was the highest in Cornezuelo (Table 2, Table 3 and Table 4; Figure 5) at the last HD, which is the usual harvesting date. This difference was mainly due to compounds **5** and **6** (oleoside/secologanoside and oleoside-11-methylester), which were much more abundant in Cornezuelo. Regarding oleuropein, the highest values were observed in Picual (13–191 mg/100 g FW) and Cornezuelo (24–160 mg/100g FW), whereas Cornicabra presented the lowest values not only of oleuropein (9–80 mg/100 g FW) but also of rutin and verbascoside.

### 3.3. Influence of Ripening on Phenolic Content

Regarding the influence of the HD (Figure 5), there was a general trend indicating that TIPC increased during the maturation process in most cases, observing the highest phenolic content in the last two HDs (corresponding approximately to maturity indexes higher than 3.5), which are the usual collecting dates for these varieties in the location areas under study. This trend was observed in both harvesting seasons. Some authors have reported decreases in total phenolics [11] or individual compounds such as oleuropein [38] along with the maturation process. Different behaviours have also been reported depending on the variety [39]. However, phenolic concentration usually increases during the maturation process until it reaches a maximum (during spotted and purple pigmentation), and then decreases [15]. In our study, we observed this trend as the ripening increased, although the decrease was not observed because the maturation indexes were not higher than five (black olive, with purple colour in less than the half of the pulp), when phenolics should decrease. Only for the last HD in Picual was the maturation index higher than 5, and there was a slight decrease in phenolic content (Table 4).

In the two first HDs there were no considerable variations between the values of TIPC for the three olive varieties. However, as fruit ripening progressed, olives from Cornezuelo and Cornicabra varieties showed increased amounts of secoiridoids and TIPC (Figure 5), whereas those from Picual did not change significantly. Among varieties, Cornezuelo and Picual presented higher TIPC values (840 mg/100 g and 670 mg/100 g, respectively) than Cornicabra variety (530 mg/100 g).

In a previous work in Memecik olive variety, the concentration of oleuropein increased during fruit ripening, from 459 mg/100 g to 994 mg/100 g [40], which is the same behaviour observed in the varieties we studied during the maturation process. This trend was also previously registered in Morisca variety, with values ranging from 195 mg/100 g FW to 264 mg/100 g FW [10]. 

In the Cornezuelo variety there was an increase in the concentration of the main secoiridoids as the olive matured. During season 2018/2019, oleoside/secologanoside, oleoside-11-methylester and oleuropein values were 25, 12.3, and 61 mg/100 g, respectively, in 1st HD, and they reached values of 220, 128 and 160 mg/100 g, respectively, in the 4th HD. This trend was also observed in the total content of secoiridoids. Similarly, the TIPC in this variety increased from 286 mg/100 g to 840 mg/100 g during olive ripening.

In Cornicabra variety, an increase in the concentrations of oleoside/secologanoside (24 mg/100 g to 170 mg/100 g) and oleoside-11-methylester (38 mg/100 g to 81 mg/100 g) was also observed during fruit ripening. Regarding the total concentration of secoiridoids and TIPC, they decreased in the 2nd HD but increased for later maturation stages.

In Picual variety, there was also an increase in the concentration of oleoside/secologanoside (38 mg/100 g to 100 mg/100 g) and oleoside-11-methylester (29 mg/100 g to 56 mg/100 g). Oleuropein reached the highest value in 3rd HD (191 mg/100 g). The behaviour of the total content of secoiridoids and the TIPC in Picual olive fruits were similar: in the two first HDs they remained constant (350–352 mg/100 g for the total content of secoiridoids and 390–392 mg/100 g for TIPC) and then increased with fruit maturation up to 550–570 mg/100 g and 640–670 mg/100 g, respectively.

To sum up, phenolic content increased during the maturation process, similarly to the trend reported in previous works, up to a maturation index of 5, at which point phenolic values are reported to decrease (an effect observed in the last HD of Picual variety). The initial hypothesis of the three olive varieties following a similar trend in phenolic concentration was confirmed, although slight variations were observed depending on the maturity index. In addition, the highest concentration of TIPC was observed in the usual olive collection dates for each olive variety (third and fourth HD).

### 3.4. Mineral Components

Although the main objective of this work was to study the influence of olive fruits maturation on the phenolic composition, we also studied the mineral components by ICP-MS. Table 5 shows the ranges of concentration levels of minerals in fruits on the analysed cultivars at different harvest dates and crops seasons evaluated. The average concentration levels obtained in each olive variety are given in Supplementary Material in Tables S6–S8. The differences observed in the different HDs and harvesting seasons are due to different humidity percentages, climatologic conditions and geographical locations. Taking into account that there were four HDs in season 2018/2019 and that phenolic concentrations were higher, the following discussion focuses on this season.

Potassium was dominant in all olive varieties; the highest concentration was found in cv. Cornicabra (7200–10,800 µg g^−1^). In Cornezuelo and Picual varieties, values of 5400–7600 µg g^−1^ and 5700–7000 µg g^−1^, respectively, were obtained. In previous studies, the concentration of K was very different depending on the olive variety: 55,000 µg g^−1^ in Leccino variety [41], and 216 µg g^−1^ in Arauco and Arbequina varieties [42]. 

Calcium was also present at high concentrations (510–810 µg g^−1^); the highest one was observed in cv. Cornicabra. Overall, these concentrations are similar to that previously found in cv. Leccino [41] and higher than those in both Arbequina and Arauco cultivars [42].

The range of copper concentrations were 1.67–3.9 µg g^−1^, 4.1–6.7 µg g^−1^ and 3.5–10 µg g^−1^ for Cornezuelo, Cornicabra and Picual varieties, respectively. These values were higher than those reported in a previous work on Arbequina, Arauco and Hojiblanca: 0.42–2.4 µg g^−1^ [42,43]. This difference is usually due to the use of copper-based fungicides to control diseases in olive trees, and the specific dose and application timing of these fungicides directly affect copper concentration in olives. Nevertheless, a Fe concentration of 2.4 µg g^−1^ was found in those varieties, similar to the obtained values in the present study (1.84–4.9 µg g^−1^).

Lower concentrations of manganese were observed in Cornezuelo, Cornicabra and Picual varieties (1.4–1.8 µg g^−1^, 1.9–3.0 µg g^−1^ and 1.16–1.40 µg g^−1^, respectively) compared to those reported in bibliography in Leccino olives (7.8 µg g^−1^) [41]. On the other hand, the maximum phosphorus concentrations for the varieties under studied were 600 µg g^−1^, 700 µg g^−1^ and 830 µg g^−1^, respectively. These maximum concentrations were reached in the 4^th^ HD and were higher than those of Arbequina and Arauco varieties (55 µg g^−1^) [42]. 

Arsenic concentration for each of the varieties remained statistically constant throughout the study (0.100–0.108 µg g^−1^, 0.095–0.103 µg g^−1^ and 0.091–0.100 µg g^−1^ in Cornezuelo, Cornicabra and Picual respectively). Similarly, cadmium values remained constant during the ripening of the olives (0.009–0.010 µg g^−1^, 0.080–0.083 µg g^−1^ and 0.079–0.084 µg g^−1^, respectively). Both toxic elements were thus present at low levels in all samples. The concentrations of Ag, Co, Cr, Mo, Pb, Sn, and V were below method detection limits in all cases.

## 4. Conclusions

In this work, we studied the effect of the ripening process on the phenolic composition and mineral content of three olive varieties: Cornezuelo, Cornicabra and Picual. This study was performed during two consecutive seasons. In all cases, most of the compounds were secoiridoids, mainly oleuropein, as expected. Cornezuelo olives had the highest total phenolic content. Phenolic concentration increased during the maturation process, up to a maturation index of approximately 5. The highest phenolic levels were observed in the third and fourth HD, which are the usual olive collection dates. The hypothesis of a similar trend in the levels of phenolics in the different olive variety was confirmed, although some differences were observed, mainly in Picual olives. However, no clear trend was observed regarding mineral composition. The sharpest increase in phenolics concentration was observed in Cornezuelo olives, which is in agreement with its latest collection date compared to the other varieties.

## Figures and Tables

**Table 1:** HPLC–ESI–MS^n^ characterization of phytochemicals in extracts of Cornezuelo, Cornicabra and Picual olive fruits in different months of season 2018/2019.

N°	t*_R_*(min)	[M-H]^−^ *m/z*	*m/z* (% Base Peak)	Assigned Identification	Cornezuelo				Cornicabra				Picual			
					1st	2nd	3rd	4th	1st	2nd	3rd	4th	1st	2nd	3rd	4th
**1**	1.8	191	MS^2^ [191]: 173 (37), 127 (7), 111 (100)	Citric acid	✓	✓	✓	✓	✓	✓	✓	✓	✓	✓	✓	✓
**2**	2.8	375	MS^2^ [375]: 213 (100), 169 (13), 151 (3), 125 (23), 107 (4) MS^3^ [375→213]: 169 (7), 125 (100)	Dihydrocominic acid				✓				✓				
**3**	3.5	315	MS^2^ [315]: 153 (100), 135 (19), 123 (22) MS^3^ [315→153]: 123 (100)	Hydroxytyrosol glucoside	✓	✓	✓	✓	✓	✓	✓	✓	✓	✓	✓	✓
**4**	4.1	407	MS^2^ [407]: 389 (100), 375 (85), 357 (71), 313 (57) MS^3^ [407→389]: 357 (62), 313 (100), 161 (22)	1-β-Glucosyl-acyclodihydroelenolic acid	✓	✓	✓	✓	✓	✓	✓	✓	✓	✓	✓	✓
**5**	7.3	389	MS^2^ [389]: 345 (100), 209 (65), 165 (25), 121 (44) MS^3^ [389→345]: 183 (51), 165 (100), 119 (48) MS^4^ [389→345→165]: 121 (100)	Oleoside/secologanoside	✓	✓	✓	✓	✓	✓	✓	✓	✓	✓	✓	✓
**6**	10.2	403	MS^2^ [403]: 223 (100), 179 (69), 143 (24), 121 (21) MS^3^ [403→223]: 191 (11), 121 (100)	Oleoside-11-methylester *	✓	✓	✓	✓	✓	✓	✓	✓	✓	✓	✓	✓
**7**	10.7	377	MS^2^ [377]: 197 (100), 153 (25) MS^3^ [377→197]: 153 (100)	Oleuropein aglycone	✓	✓	✓	✓	✓	✓	✓	✓	✓	✓	✓	✓
**8**	13.4	337	MS^2^ [337]: 178 (41), 115 (6), 114 (100)	Unknown					✓	✓	✓	✓		✓	✓	
**9**	13.6	403	MS^2^ [403]: 371 (100), 223 (70), 179 (78), 121 (13)	Elenolic acid glucoside	✓	✓	✓	✓					✓	✓	✓	
**10**	17.6	525	MS^2^ [525]: 481 (29), 319 (23), 301 (23), 195 (100) MS^3^ [525→195]: 193 (100), 192 (50)	Dimethyl-oleuropein glucoside					✓	✓						
**11**	18.1	701	MS^2^ [701]: 539 (34), 469 (29), 385 (17), 315 (100) MS^3^ [701→315]: 153 (100), 123 (24) MS^4^ [701→315→153]: 123 (100)	Hydroxytyrosol glucoside derivative			✓	✓		✓	✓	✓				
**12**	19.4	609	MS^2^ [609]: 301 (100) MS^3^ [609→301]: 179 (100), 151 (70) MS^4^ [609→301→179]: 151 (100)	Rutin *	✓	✓	✓	✓	✓	✓	✓	✓	✓	✓	✓	✓
**13**	20.3	623	MS^2^ [623]: 461 (100) MS^3^ [623→461]: 315 (58), 135 (100)	Verbascoside *	✓		✓	✓	✓	✓	✓	✓	✓	✓	✓	✓
**14**	20.8	447	MS^2^ [447]: 285 (100) MS^3^ [447→285]: 241 (90), 175 (100)	Luteolin-*O*-hexoside	✓	✓	✓	✓	✓	✓	✓	✓	✓	✓	✓	✓
**15**	21.2	543	MS^2^ [543]: 525 (100), 513 (31), 389 (15)MS^3^ [543→525]: 389 (100), 357 (73), 313 (83) MS^4^ [543→525→389]: 357 (59), 313 (100)	Dihydrooleuropein	✓	✓	✓	✓	✓	✓	✓	✓	✓	✓	✓	✓
**16**	22.1	701	MS^2^ [701]: 539 (100), 377 (82), 307 (54), 275 (46) MS^3^ [701→539]: 507 (32), 377 (100), 307 (94), 275 (73) MS^4^ [701→539→377]: 275 (100)	Oleuropein glucoside	✓	✓	✓	✓	✓	✓	✓	✓	✓	✓	✓	✓
**17**	23.1	701	MS^2^ [701]: 539 (100), 377 (43), 307 (42), 275 (34) MS^3^ [701→539]: 377 (54), 307 (50), 275 (100) MS^4^ [701→539→275]: 139 (100), 113 (86)	Oleuropein glucoside	✓	✓	✓	✓		✓				✓	✓	
**18**	24.3	551	MS^2^ [551]: 507 (100), 341 (48), 281 (37), 251 (39), 179 (40) MS^3^ [551→507]: 341 (34), 179 (34), 161 (100)	6′-β-hexopyranosyloleoside	✓	✓	✓	✓	✓	✓	✓	✓	✓	✓	✓	✓
**19**	25.4	377	MS^2^ [377]: 307 (100), 275 (72) MS^3^ [377→307]: 275 (100), 139 (22) MS^4^ [377→307→275]: 113 (100)	Oleuropein aglycone						✓	✓			✓	✓	
**20**	26.0	569	MS^2^ [569]: 537 (90), 403 (100), 223 (25) MS^3^ [569→403]: 371 (10), 223 (100), 179 (81), 143 (28) MS^4^ [569→403→223]: 121 (100), 101 (24)	Elenolic acid glucoside derivative				✓			✓	✓			✓	
**21**	27.0	539	MS^2^ [539]: 377 (100), 307 (57), 275 (72) MS^3^ [539→377]: 307 (100), 275 (87), 149 (8) MS^4^ [539→377→307]: 275 (100)	Oleuropein *	✓	✓	✓	✓	✓	✓	✓	✓	✓	✓	✓	✓
**22**	28.3	535	MS^2^ [535]: 491 (100), 265 (36), 235 (28) MS^3^ [535→491]: 345 (46), 265 (46), 145 (100)	Comselogoside isomer	✓	✓	✓	✓	✓	✓	✓	✓	✓	✓	✓	✓
**23**	29.4	539	MS^2^ [539]: 377 (71), 307 (85), 275 (100)	Oleuropein isomer	✓	✓	✓	✓	✓	✓	✓	✓	✓	✓	✓	✓
**24**	30.1	535	MS^2^ [535]: 491 (100), 265 (60), 235 (44), 209 (37) MS^3^ [535→491]: 345 (25), 235 (46), 206 (19), 145 (100) MS^4^ [535→491→145]: 143 (100)	Comselogoside isomer	✓	✓	✓	✓		✓	✓	✓	✓			
**25**	30.6	473	MS^2^ [473]: 358 (29), 195 (100), 178 (7) MS^3^ [473→195]: 135 (100)	Unknown	✓	✓	✓		✓	✓	✓	✓	✓	✓	✓	
**26**	32.2	523	MS^2^ [523]: 361 (100), 291 (59), 259 (47) MS^3^ [523→361]: 291 (100), 259 (77), 223 (12)	Ligstroside	✓	✓	✓	✓	✓	✓	✓	✓	✓	✓	✓	✓
**27**	36.3	557	MS^2^ [557]: 513 (100), 345 (46), 209 (66), 185 (56) MS^3^ [557→513]: 345 (100), 199 (19), 185 (59) MS^4^ [557→513→345]: 183 (100)	Oleoside/secologanoside derivative	✓	✓	✓	✓	✓	✓			✓	✓	✓	✓

* Identified with analytical standard.

**Table 2:** Quantification of phenolic compounds in olive fruits (cv. Cornezuelo) at different harvest times and harvest seasons.

Cornezuelo				2017/2018			2018/2019			
				2nd HD	3rd HD	4th HD	1st HD	2nd HD	3rd HD	4th HD
MI				2	3.5	4	1	1.5	3	4
**(Seco)iridoids**										
**5**	Oleoside/secologanoside			20 ± 2 ^b^	26 ± 3 ^b^	100 ± 10 ^a^	25 ± 3 ^c^	38.4 ± 0.3 ^c^	102 ± 5 ^b^	220 ± 20 ^a^
**6**	Oleoside-11-methylester			0.48 ± 0.03 ^b^	6.1 ± 0.5 ^b^	65 ± 6 ^a^	12.3 ± 0.7 ^d^	22 ± 2 ^c^	70 ± 2 ^b^	128 ± 5 ^a^
**7**	Oleuropein aglycone			3.62 ± 0.08	4.0 ± 0.5	-	2.2 ± 0.1 ^b^	2.4 ± 0.1 ^b^	6.3 ± 0.1 ^a^	-
**9**	Elenolic acid glucoside			11 ± 2 ^b^	7.8 ± 0.5 ^b^	47 ± 1 ^a^	16 ± 2 ^c^	14.19 ± 0.05 ^c^	69 ± 5 ^b^	88 ± 7 ^a^
**15**	Dihydrooleuropein			-	-	-	4.6 ± 0.5 ^a^	4.3 ± 0.5 ^a^	4.4 ± 0.5 ^a^	4.6 ± 0.4 ^a^
**16**	Oleuropein glucoside			7.8 ± 0.3 ^b^	11.4 ± 0.2 ^a^	6.8 ± 0.7 ^b^	6.3 ± 0.7 ^b^	6.5 ± 0.3 ^b^	9.2 ± 0.9 ^a^	7.7 ± 0.3 ^ab^
**17**	Oleuropein glucoside			2.3 ± 0.3 ^c^	4.0 ± 0.4 ^b^	9.3 ± 0.9 ^a^	2.63 ± 0.01 ^c^	-	11.7 ± 0.6 ^b^	14 ± 1 ^a^
**18**	6′-β-hexopyranosyloleoside			35 ± 3 ^ab^	39 ± 4 ^a^	30.8 ± 0.3 ^b^	30 ± 3 ^a^	32.5 ± 0.4 ^a^	32 ± 2 ^a^	32 ± 1 ^a^
**20**	Elenolic acid glucoside derivative			-	3.6 ± 0.1	24 ± 2	-	-	-	13.2 ± 0.6
**21**	Oleuropein			24 ± 1 ^c^	60 ± 3 ^b^	88 ± 5 ^a^	61 ± 5 ^c^	78 ± 6 ^c^	131 ± 8 ^b^	160 ± 10 ^a^
**22**	Comselogoside isomer			99 ± 8 ^ab^	110 ± 10 ^a^	81.9 ± 0.6 ^b^	62 ± 5 ^b c^	64.7 ± 0.1 ^ab^	70 ± 1 ^a^	55 ± 2 ^c^
**23**	Oleuropein isomer			37 ± 4	42 ± 4	30.3 ± 0.2	34 ± 3 ^a^	38 ± 2 ^a^	35 ± 1 ^a^	28 ± 2 ^b^
**24**	Comselogoside isomer			5.6 ± 0.6 ^b^	11 ± 1 ^a^	9.4 ± 0.9 ^a^	5.3 ± 0.6 ^b^	5.0 ± 0.2 ^b^	5.1 ± 0.1 ^b^	6.4 ± 0.3 ^a^
**26**	Ligstroside			2.7 ± 0.3 ^b^	6.6 ± 0.7 ^a^	7.2 ± 0.8 ^a^	7.9 ± 0.9 ^b^	7.6 ± 0.6 ^b^	19 ± 2 ^a^	10 ± 1 ^b^
**27**	Oleoside/secologanoside derivative			16.53 ± 0.04 ^a^	13 ± 1 ^b^	15 ± 1 ^ab^	9.2 ± 0.9 ^c^	8.808 ± 0.003 ^c^	17 ± 2 ^a^	13.0 ± 0.3 ^b^
**Total**				270 ± 10 ^c^	340 ± 10 ^b^	510 ± 10 ^a^	278 ± 9 ^d^	322 ± 7 ^c^	580 ± 10 ^b^	780 ± 20 ^a^
**Other compounds**										
**3**		Hydroxytyrosol glucoside		2.4 ± 0.3 ^c^	12 ± 1 ^b^	30 ± 3 ^a^	3.15 ± 0.05 ^d^	6.5 ± 0.4 ^c^	10.8 ± 0.7 ^b^	35.9 ± 0.9 ^a^
**11**		Hydroxytyrosol glucoside derivative		-	3.9 ± 0.4	9.5 ± 0.2	-	-	4.6 ± 0.4	12.2 ± 0.6
**12**		Rutin		19 ± 1 ^a^	17 ± 2 ^a^	5.7 ± 0.5 ^b^	4.5 ± 0.5 ^b^	4.7 ± 0.5 ^b^	10.9 ± 0.9 ^a^	3.3 ± 0.3 ^b^
**13**		Verbascoside		0.026 ± 0.002 ^c^	0.26 ± 0.03 ^b^	2.47 ± 0.01 ^a^	0.040 ± 0.003 ^c^	-	2.38 ± 0.08 ^b^	6.39 ± 0.09 ^a^
**14**		Luteolin-*O*-hexoside		10.9 ± 0.6 ^b^	18 ± 2 ^a^	10.7 ± 0.1 ^b^	-	1.5 ± 0.1 ^c^	9.2 ± 0.8 ^a^	6.9 ± 0.5 ^b^
**Total**				32 ± 1 ^c^	51 ± 3 ^b^	58 ± 3 ^a^	7.7 ± 0.5 ^d^	12.7 ± 0.6 ^c^	38 ± 1 ^b^	65 ± 1 ^a^
**TIPC**				300 ± 10 ^c^	390 ± 10 ^b^	570 ± 10 ^a^	286 ± 9 ^d^	335 ± 7 ^c^	620 ± 10 ^b^	840 ± 20 ^a^

Values (mg/100 g fresh weight) are mean ± SD of three parallel measurements. Different superscripts (^a^, ^b^, ^c^ and ^d^) indicate significant differences in the extracts (*p* < 0.05). MI: Maturity Index.

**Table 3:** Quantification of phenolic compounds in olive fruits (cv. Cornicabra) at different harvest times and harvest seasons.

Cornicabra				2017/2018			2018/2019			
				2nd HD	3rd HD	4th HD	1st HD	2nd HD	3rd HD	4th HD
MI				1	3	4	1	3	4.5	4.75
**(Seco)iridoids**										
**5**	Oleoside/secologanoside			7.4 ± 0.7 ^c^	81 ± 4 ^a^	51 ± 4 ^b^	24 ± 2 ^d^	55 ± 5 ^c^	84 ± 3 ^b^	170 ± 10 ^a^
**6**	Oleoside-11-methylester			-	53 ± 4	25 ± 2	38 ± 3 ^c^	40 ± 3 ^c^	58 ± 4 ^b^	81 ± 6 ^a^
**7**	Oleuropein aglycone			2.10 ± 0.07	-	-	1.71 ± 0.03 ^a^	0.93 ± 0.09 ^c^	1.49 ± 0.06 ^b^	-
**10**	Dimethyl-oleuropein glucoside			-	-	-	11 ± 1	-	-	-
**15**	Dihydrooleuropein			-	-	-	2.4 ± 0.3 ^b^	1.5 ± 0.1 ^c^	1.8 ± 0.2 ^bc^	3.1 ± 0.3 ^a^
**16**	Oleuropein glucoside			3.3 ± 0.1 ^b^	3.34 ± 0.02 ^b^	9.0 ± 0.9 ^a^	3.7 ± 0.3 ^c^	7.6 ± 0.2 ^a^	6.2 ± 0.6 ^b^	6.9 ± 0.6 ^ab^
**17**	Oleuropein glucoside			-	72 ± 2	30 ± 3	-	-	-	-
**18**	6′-β -Hexopyranosyloleoside			-	-	-	9.8 ± 0.3 ^c^	6.2 ± 0.2 ^d^	13 ± 1 ^b^	16 ± 1 ^a^
**20**	Elenolic acid glucoside derivative			-	82 ± 2	28 ± 2	-	-	5.4 ± 0.3	60 ± 6
**21**	Oleuropein			9.3 ± 0.8 ^b^	80 ± 5 ^a^	75 ± 4 ^a^	72 ± 5 ^a^	62 ± 4 ^ab^	67 ± 4 ^ab^	56 ± 4 ^b^
**22**	Comselogoside isomer			-	-	-	26 ± 2 ^a^	15.3 ± 0.7 ^b^	27 ± 2 ^a^	29 ± 1 ^a^
**23**	Oleuropein isomer			30 ± 3	-	34 ± 1	39 ± 4 ^a^	18.1 ± 0.7 ^c^	27 ± 3 ^b^	12 ± 1 ^c^
**24**	Comselogoside isomer			-	-	-	-	-	-	3.6 ± 0.2
**26**	Ligstroside			1.7 ± 0.1 ^b^	8.2 ± 0.9 ^a^	7.0 ± 0.8 ^a^	21 ± 2 ^a^	7.6 ± 0.8 ^c^	16 ± 1 ^b^	9.2 ± 0.5 ^c^
**27**	Oleoside/secologanoside derivative			2.4 ± 0.2	-	-	7.0 ± 0.6	-	-	-
**Total**				56 ± 3 ^c^	380 ± 8 ^a^	259 ± 7 ^b^	256 ± 8 ^c^	214 ± 7 ^d^	307 ± 8 ^b^	450 ± 20 ^a^
**Other compounds**										
**3**		Hydroxytyrosol glucoside		-	-	7.5 ± 0.8	2.3 ± 0.1 ^d^	9.0 ± 0.9 ^c^	33 ± 3 ^b^	63 ± 4 ^a^
**11**		Hydroxytyrosol glucoside derivative		-	3.6 ± 0.1	5.4 ± 0.4	-	2.0 ± 0.2 ^c^	3.4 ± 0.4 ^b^	5.1 ± 0.1 ^a^
**12**		Rutin		8.7 ± 0.5 ^a^	6.8 ± 0.4 ^b^	4.1 ± 0.4 ^c^	7.9 ± 0.8 ^a^	3.2 ± 0.4 ^c^	6.0 ± 0.7 ^b^	4.8 ± 0.5 ^b c^
**13**		Verbascoside		-	-	1.54 ± 0.08	0.17 ± 0.01 ^c^	0.27 ± 0.01 ^c^	3.08 ± 0.07 ^a^	1.32 ± 0.08 ^b^
**14**		Luteolin-*O*-hexoside		2.18 ± 0.02 ^c^	9.2 ± 0.3 ^b^	11.1 ± 0.9 ^a^	-	2.21 ± 0.05 ^c^	6.6 ± 0.3 ^b^	7.2 ± 0.2 ^a^
**Total**				10.9 ± 0.5 ^c^	19.6 ± 0.5 ^b^	30 ± 1 ^a^	10.4 ± 0.8 ^c^	17 ± 1 ^c^	52 ± 3 ^b^	81 ± 4 ^a^
**TIPC**				67 ± 3 ^c^	400 ± 8 ^a^	289 ± 7 ^b^	266 ± 8 ^c^	231 ± 7 ^d^	359 ± 9 ^b^	530 ± 20 ^a^

Values (mg/100 g fresh weight) are mean ± SD of three parallel measurements. Different superscripts (^a^, ^b^, ^c^ and ^d^) indicate significant differences in the extracts (*p* < 0.05). MI: Maturity Index.

**Table 4:** Quantification of phenolic compounds in olive fruits (cv. Picual) at different harvest times and harvest seasons.

Picual				2017/2018			2018/2019			
				2nd HD	3rd HD	4th HD	1st HD	2nd HD	3rd HD	4th HD
MI				2	3.75	4.25	1	2.75	5	5.75
**(Seco)iridoids**										
**5**	Oleoside/secologanoside			-	8.8 ± 0.8	25.1 ± 6	38 ± 4 ^c^	36.6 ± 0.4 ^c^	74 ± 7 ^b^	100 ± 10 ^a^
**6**	Oleoside-11-methylester			-	1.4 ± 0.2	10.9 ± 0.9	29 ± 3 ^b^	27 ± 2 ^b^	54 ± 4 ^a^	56 ± 4 ^a^
**7**	Oleuropein aglycone			2.2 ± 0.1 ^b^	3.1 ± 0.5 ^a^	2.6 ± 0.3 ^ab^	3.7 ± 0.4 ^b^	3.5 ± 0.1 ^b^	6.0 ± 0.5 ^a^	4.02 ± 0.04 ^b^
**9**	Elenolic acid glucoside			-	-	-	10.3 ± 0.2	-	-	-
**15**	Dihydrooleuropein			6.0 ± 0.6 ^a^	5.4 ± 0.5 ^a^	2.8 ± 0.3 ^b^	4.1 ± 0.3 ^b^	4.2 ± 0.4 ^b^	4.5 ± 0.5 ^ab^	5.5 ± 0.5 ^a^
**16**	Oleuropein glucoside			4.5 ± 0.6 ^b^	3.0 ± 0.4 ^b^	9.3 ± 0.9 ^a^	5.0 ± 0.4 ^b^	6.4 ± 0.7 ^ab^	7.5 ± 0.7 ^a^	7.7 ± 0.2 ^a^
**17**	Oleuropein glucoside			2.5 ± 0.3 ^a^	2.5 ± 0.3 ^a^	2.0 ± 0.1 ^a^	-	-	4.0 ± 0.4	-
**18**	6′-β-hexopyranosyloleoside			23 ± 1 ^b^	30 ± 3 ^a^	25 ± 1 ^b^	31.9 ± 0.2 ^b^	32 ± 3 ^b^	41 ± 1 ^a^	40 ± 2 ^a^
**20**	Elenolic acid glucoside derivative			-	21.1 ± 0.5	15.4 ± 0.6	-	-	12 ± 1	-
**21**	Oleuropein			13.1 ± 0.8 ^c^	16.5 ± 0.9 ^b^	44 ± 2 ^a^	107 ± 5 ^c^	85 ± 5 ^d^	191 ± 9 ^a^	152 ± 7 ^b^
**22**	Comselogoside isomer			42 ± 4 ^ab^	46 ± 4 ^a^	37 ± 2 ^b^	42.4 ± 0.7 ^b^	46 ± 3 ^b^	53 ± 2 ^a^	45 ± 3 ^b^
**23**	Oleuropein isomer			-	-	-	52 ± 5 ^c^	91 ± 9 ^b^	86 ± 8 ^b^	114 ± 3 ^a^
**24**	Comselogoside isomer			3.5 ± 0.4 ^a^	3.7 ± 0.4 ^a^	4.4 ± 0.5 ^a^	2.6 ± 0.3	-	-	-
**26**	Ligstroside			-	-	2.6 ± 0.3	17 ± 2 ^b^	11 ± 1 ^c^	23 ± 2 ^a^	19 ± 2 ^ab^
**27**	Oleoside/secologanoside derivative			-	-	2.8 ± 0.3	9.05 ± 0.07 ^b^	8.1 ± 0.8 ^b^	10.9 ± 0.4 ^a^	8.410 ± 0.002 ^b^
**Total**				97 ± 4 ^c^	142 ± 5 ^b^	184 ± 7 ^a^	352 ± 9 ^b^	350 ± 10 ^b^	570 ± 20 ^a^	550 ± 10 ^a^
**Other compounds**										
**3**		Hydroxytyrosol glucoside		9.3 ± 0.8 ^a^	11 ± 1 ^a^	10.5 ± 0.6 ^a^	20 ± 2 ^b^	14 ± 1 ^c^	21 ± 2 ^b^	31 ± 2 ^a^
**12**		Rutin		8.3 ± 0.2 ^b^	8.0 ± 0.3 ^b^	10.3 ± 0.5 ^a^	7.9 ± 0.8 ^c^	9.2 ± 0.9 ^c^	32 ± 3 ^a^	16.5 ± 0.2 ^b^
**13**		Verbascoside		26 ± 1 ^a^	23 ± 1 ^b^	17 ± 1 ^c^	9.2 ± 0.8 ^b^	5.8 ± 0.4 ^c^	10.1 ± 0.8 ^b^	17.8 ± 0.8 ^a^
**14**		Luteolin-*O*-hexoside		4.9 ± 0.3 ^c^	20 ± 1 ^b^	37 ± 1 ^a^	2.9 ± 0.3 ^d^	11 ± 1 ^c^	37 ± 4 ^a^	25 ± 2 ^b^
**Total**				49 ± 1 ^c^	62 ± 2 ^b^	75 ± 2 ^a^	40 ± 2 ^c^	40 ± 2 ^c^	100 ± 5 ^a^	90 ± 3 ^b^
**TIPC**				146 ± 4 ^c^	204 ± 5 ^b^	259 ± 7 ^a^	392 ± 9 ^b^	390 ± 10 ^b^	670 ± 20 ^a^	640 ± 10 ^a^

Values (mg/100 g fresh weight) are mean ± SD of three parallel measurements. Different superscripts (^a^, ^b^, ^c^ and ^d^) indicate significant differences in the extracts (*p* < 0.05). MI: Maturity Index.

**Table 5:** Concentrations of mineral in foods of the analysed olive cultivars at two different harvest seasons.

	CORNEZUELO		CORNICABRA		PICUAL	
Element	2017/2018	2018/2019	2017/2018	2018/2019	2017/2018	2018/2019
As	0.231–0.260	0.100–0.108	0.224–0.255	0.095–0.103	0.23–0.26	0.091–0.100
Ba	0.24–0.27	-	0.874–0.950	0.56–0.83	0.35–0.54	0.099–0.122
Ca	370–620	260–690	590–980	440–810	340–700	187–510
Cd	0.145–0.160	0.009–0.010	0.138–0.157	0.080–0.083	0.15–0.16	0.079–0.084
Cu	1.4–2.0	1.67–3.9	1.47–3.30	4.1–6.7	2.38–4.35	3.5–10.0
Fe	10.0	Detected *	Detected *	2.5–4.9	Detected *	1.84–2.5
K	4600–5700	5400–7600	7000–11,400	7200–10,800	5300–10,100	5700–7000
Mg	130–180	150–210	126–209	160–202	109–257	119–152
Mn	1.30–1.86	1.4–1.8	1.42–2.30	1.9–3.0	1.31–2.00	1.16–1.40
Mo	0.122–0.139	-	0.131–0.168	-	0.13–0.15	-
Na	-	5.1	-	3.4–6.3	-	2.90–4.05
Ni	Detected *	0.046–0.058	Detected *	0.236–0.510	Detected *	0.062–0.180
P	330–430	410–600	200–380	320–700	260–458	380–830
Sb	0.075–0.088	0.071–0.089	0.073–0.084	-	0.080–0.083	-
Sn	Detected *	-	Detected *	-	Detected *	-
Ti	-	-	-	0.072–0.075	-	0.13
Zn	2.8–3.7	2.48–3.20	2.6–2.8	3.22–5.54	2.53–4.30	1.70–2.53

Data are expressed as ranges of concentrations (µg g^−1^ fresh weight) of three (2017/2018 season) or four (2018/2019 season) harvest times. * Concentrations between detection and quantitation limits.

## Data Availability

Data available on request.

## Supplementary Materials

The following are available online at https://www.mdpi.com/2304-8158/10/2/380/s1. Chromatographic conditions; ICP-MS characteristics; Table S1: ICP-MS operating conditions; Table S2: Analysis of the cranberry certified reference material (NIST-3281); Table S3: Method detection limits for the analysed elements in ICP-MS; Table S4: Digestion procedure conditions; Table S5: Humidity percentages of all the olive samples analysed; Tables S6–S8: Average concentration levels of minerals and trace elements in the analysed Cornezuelo, Cornicabra and Picual olives in different months of two seasons; Figure S1: Recovery yields with the olives of the sample “Cornicabra 1st HD”.

## Abbreviations

1st	October
2nd	November
3rd	December
4th	January
DE	Dried extract
FW	Fresh weight
HD	Harvest date
*m*/*z*	Mass to charge ratio
SD	Standard deviation
TIPC	Total individual phenolic content
t_R_	Retention time
