# Supplementary material for: Effect of Ripening on the Phenolic Composition and Mineral Content of Three Varieties of Olive Fruits
Source: Foods. 2021 Feb 9;10(2):380. doi: 10.3390/foods10020380 (PMC7919262; doi:10.3390/foods10020380)

Effect of Ripening on the Phenolic Composition and Mineral Content of Three Varieties of Spanish Olive Fruits

María del Pilar Fernández-Poyatos, Eulogio J. Llorent-Martínez and Antonio Ruiz-Medina*

Department of Physical and Analytical Chemistry, Faculty of Experimental Sciences, University of Jaén, Campus Las Lagunillas, E-23071 Jaén, Spain

*Correspondence: anruiz@ujaen.es; Tel.: +34 953 212759

Supplementary Material

Chromatographic Conditions

Chromatographic conditions are based on previous work by our research group [1]. In these analyses, 5 mg of DE were re-dissolved in 1 mL of MeOH:H_2O (80:20; v:v), filtered through 0.45 µm filters, and 10 µL of sample was analyzed. The HPLC system was an Agilent Series 1100, composed of a vacuum degasser, an autosampler, a binary pump and a G1315B diode array detector (Agilent Technologies; Santa Clara, CA, USA). We used a reversed phase Luna Omega Polar C18 analytical column of 150 x 3.0 mm and 5 µm particle size (Phenomenex; Torrance, CA, USA) and a Polar C18 Security Guard cartridge (Phenomenex) of 4 x 3.0 mm. The best separation was achieved with a mobile phase of H_2O:HCOOH (100:0.1, v/v) and CH_3CN. The following program was used: a) initial mobile phase, 10% CH_3CN; b) linear increase from 10% to 25% CH_3CN (0-25 min); c) 25% CH_3CN (25-30 min); d) linear increase from 25% to 50% CH_3CN (30-40 min); e) linear increase from 50% to 100% CH_3CN (40-42 min); f) 100% CH_3CN (42-47 min). Then, CH_3CN percentage was returned to the initial mobile phase, with a 7 min stabilization time. The flow rate was 0.4 ml min^{-1}.

The HPLC system was connected to an ion trap mass spectrometer (Esquire 6000, Bruker Daltonics, Billerica, MA, USA) equipped with an electrospray interface. The scan range was at m/z 100–1200 with a speed of 13,000 Da/s. The ESI conditions were: drying gas (N_2) flow rate and temperature, 10 L/min and 365 ℃, respectively; nebulizer gas (N_2) pressure, 50 psi; capillary voltage, 4500 V; capillary exit voltage, -117.3 V. We used the auto MS^n mode for the acquisition of MS^n data, with isolation width of 4.0 m/z, and fragmentation amplitude of 0.6 V (MS^n up to MS^4). The negative ion mode was used in all cases.

Calibration curves for hydroxytyrosol, luteolin, oleuropein, oleoside-11-methylester, rutin and verbascoside were prepared in the range of 0.5–100 µg mL^{-1}. Chromatograms were recorded at 240, 280 and 350 nm for secoiridoids, hydroxytyrosol and luteolin, respectively. For oleoside-11-methylester, oleuropein, rutin and verbascoside, the instrument was operated in product ion scan

MS/MS mode with MS/MS transitions 403→223, 539→377, 609→301 and 623→461, respectively, and voltage amplitudes between 0.4 and 0.7 V.

ICP-MS Characteristics

ICP-MS conditions are based on previous work by our research group [1]. An Agilent 7900 (Agilent Technologies, CA, USA) equipped with a Peltier-cooled quartz spray chamber (Scott type), a quartz torch (2.5 mm i.d.), a low flow concentric nebulizer (0.2 mL/min) and high efficiency “MicroMist”, were used. The instrument is equipped with a discrete sampling system (ISIS3), an autosampler (SPS4), a sample introduction system (UHMI), ion lenses off-axis, a high-frequency quadrupole (3 MHz), a radiofrequency generator (27 MHz), an octopolar collision-reaction cell (ORS system), and a secondary electron multiplier detector. The following solutions were also used: 100 µg mL^{-1} multi-element standard (SCP Science, Paris, France), 1000 µg mL^{-1} P solution (Sigma-Aldrich), 1000 µg mL^{-1} Sn (High-Purity Standards, Charleston, SC, USA) and an internal standard kit (Sc, Ge, Rh and Ir) from ISC Science (Oviedo, Spain).

We studied the inorganic content of Cornezuelo, Cornicabra and Picual olives by ICP-MS. For the validation of the method, we carried out recovery studies of the sample “Cornicabra 1st HD”, spiked at different concentrations, as well as the analysis of a certified reference material (cranberry SRM 3281). Recovery studies were performed for all the analyzed elements, observing recovery yields between 91% and 116%, with standard deviations lower than 8%. The results obtained in the analysis of the certified reference material was also satisfactory; the student’s t statistical test (p=0.05) was performed, observing no significant differences between the certified and obtained values. These results, ICP-MS operating conditions and the method detection limits of each element are shown in the following Tables (S1 to S3) and Figure S1.

Reference

1. Fernández-Poyatos, M.D.P.; Ruiz-Medina, A.; Llorent-Martínez, E.J. Phytochemical profile and mineral content of Royal variety olive fruits. Influence of the ripening stage. *J. Food Compos. Anal.* **2021**, *95*, 103671, doi:10.1016/j.jfca.2020.103671.

Table S1. ICP-MS operating conditions.

Plasma conditions: Tune Parameters	
RF Power	1550 W
RF Matching	1.80 V
Sample Depth	10.0 mm
Nebulizer Gas	0.99 L/min
Gas Switch	Makeup Gas
Makeup/Dilution Gas	0.00 L/min
Option Gas	0.0 %
Nebulizer Pump	0.10 rps
S/C Temp	2 ℃
Plasma Gas	15.0 L/min
Auxiliary Gas	0.90 L/min
**Torch conditions: Hardware Settings**	
Torch H	0.1 mm
Torch V	0.2 mm

Table S2. Analysis of the cranberry certified reference material (NIST-3281).

Element	Certified value[a] (mg kg^{-1})	Observed value[b] (mg kg^{-1})	t_{calc}[c]
Ca	528 ± 7	503 ± 10	3.01
Cu	3.52 ± 0.09	3.36 ± 0.05	2.65
Fe	27.7 ± 0.7	25.6 ± 0.9	2.74
Mg	446 ± 4	453 ± 5	1.63
Mn	21.9 ± 0.2	21.8 ± 0.3	0.40
P	835 ± 17	833 ± 18	0.12
K	8020 ± 130	7950 ± 160	0.51
Na	259 ± 3	264 ± 5	1.24
Zn	6.9 ± 0.2	6.5 ± 0.1	3.10

[a]n=2; [b]n=3; [c]p=0.05, $t_{Student}$ (theoretical value)=3.182.
Results are given as mean ± standard deviation (n=2 in certified values; n=3 in observed values).

Table S3. Method detection limits for the analyzed elements in ICP-MS.

Element	MDL (μg g^{-1})	Element	MDL (μg g^{-1})
Ag	1.7	Mn	0.032
Al	0.82	Mo	0.004
As	0.002	Na	0.8
Ba	0.007	Ni	0.012
Ca	6.2	P	1.35
Cd	0.002	Pb	0.01
Co	0.006	Sb	0.004
Cr	0.02	Se	0.32
Cu	0.08	Sn	0.06
Fe	0.5	Ti	0.018
K	5	V	0.004
Mg	0.04	Zn	0.2

Table S4. Digestion procedure conditions.

Power level (W)	Power (%)	Ramp time (min)	Temperature (℃)	Hold time (℃)
1600	100	5	100	7
1600	100	5	125	7
1600	100	5	150	7

Table S5. Humidity percentages of all the olive samples analyzed.

Variety	Season	Humidity (%)			
		1st HD	2nd HD	3rd HD	4th HD
Cornezuelo	2017/2018	-	56	46	42
	2018/2019	60	58	44	38
Cornicabra	2017/2018	-	65	46	49
	2018/2019	68	59	54	29
Picual	2017/2018	-	67	49	48
	2018/2019	64	56	39	36

Table S6. Average concentration levels of minerals and trace elements in the analyzed Cornezuelo olives in different months of two seasons; expressed in µg g^{-1} fresh weight. Results are given as mean ± standard deviation (n=3).

Cornezuelo	**2017/2018**			**2018/2019**			
**Element**	**2nd HD**	**3rd HD**	**4th HD**	**1st HD**	**2nd HD**	**3rd HD**	**4th HD**
As	0.26 ± 0.01ᵃ	0.231 ± 0.009ᵇ	0.241 ± 0.004ᵃᵇ	0.113 ± 0.001ᵃ	0.101 ± 0.005ᵃ	0.10 ± 0.01ᵃ	0.108 ± 0.001ᵃ
Ba	0.27 ± 0.03ᵃ	0.24 ± 0.03ᵃ	0.25 ± 0.03ᵃ	-	-	-	-
Ca	620 ± 60ᵃ	590 ± 50ᵃ	370 ± 40ᵇ	260 ± 20ᵇ	350 ± 30ᵇ	690 ± 70ᵃ	260 ± 30ᵇ
Cd	0.160 ± 0.006ᵃ	0.145 ± 0.007ᵇ	0.150 ± 0.003ᵃᵇ	0.010 ± 0.001	0.009 ± 0.001	Detected*	Detected*
Cu	1.68 ± 0.17ᵃᵇ	2.0 ± 0.3ᵃ	1.4 ± 0.2ᵇ	1.67 ± 0.01ᶜ	3.2 ± 0.1ᵇ	1.9 ± 0.2ᶜ	3.9 ± 0.4ᵃ
Fe	-	Detected*	10.0 ± 0.1	Detected*	Detected*	Detected*	Detected*
K	4600 ± 400ᵃ	5700 ± 400ᵃ	5100 ± 600ᵃ	5400 ± 300ᵇ	5400 ± 100ᵇ	7400 ± 100ᵃ	7600 ± 200ᵃ
Mg	180 ± 10ᵃ	132 ± 7ᵇ	130 ± 20ᵇ	165.8 ± 0.3ᵇ	210 ± 7ᵃ	210 ± 20ᵃ	150 ± 10ᵇ
Mn	1.86 ± 0.06ᵃ	1.6 ± 0.2ᵃᵇ	1.3 ± 0.1ᵇ	1.510 ± 0.003ᵃᵇ	1.7 ± 0.2ᵃᵇ	1.8 ± 0.2ᵃ	1.4 ± 0.1ᵇ
Mo	0.139 ± 0.005ᵃ	0.122 ± 0.003ᵇ	0.127 ± 0.006ᵃᵇ	-	-	-	-
Na	-	-	-	5.1 ± 0.6	Detected*	Detected*	Detected*
Ni	-	Detected*	-	0.046 ± 0.002ᵇ	0.058 ± 0.006ᵃ	Detected*	0.051 ± 0.005ᵃᵇ
P	430 ± 50ᵃ	410 ± 40ᵃ	330 ± 40ᵃ	410 ± 20ᵇ	460 ± 40ᵇ	450 ± 10ᵇ	600 ± 40ᵃ
Sb	0.088 ± 0.001ᵃ	0.075 ± 0.002ᵇ	0.078 ± 0.001ᵇ	0.089 ± 0.004ᵃ	0.073 ± 0.004ᵇ	0.071 ± 0.005ᵇ	0.074 ± 0.001ᵇ
Sn	Detected*	Detected*	Detected*	-	-	-	-
Zn	3.7 ± 0.3ᵃ	3.6 ± 0.4ᵃ	2.8 ± 0.2ᵇ	2.5 ± 0.1ᵇ	3.2 ± 0.3ᵃ	2.48 ± 0.07ᵇ	2.5 ± 0.2ᵇ

*Concentrations between detection and quantitation limits.
Different superscripts ([a], [b] and [c]) indicate significant differences in the extracts ($p<0.05$).

Table S7. Average concentration levels of minerals and trace elements in the analyzed Cornicabra olives in different months of two seasons; expressed in µg g^{-1} fresh weight. Results are given as mean ± standard deviation (n=3).

Cornicabra	**2017/2018**			**2018/2019**			
**Element**	**2nd HD**	**3rd HD**	**4th HD**	**1st HD**	**2nd HD**	**3rd HD**	**4th HD**
As	0.248 ± 0.005^{a}	0.255 ± 0.007^{a}	0.224 ± 0.009^{b}	0.096 ± 0.003^{a}	0.095 ± 0.005^{a}	0.096 ± 0.002^{a}	0.103 ± 0.007^{a}
Ba	0.94 ± 0.08^{a}	0.874 ± 0.007^{a}	0.95 ± 0.09^{a}	0.80 ± 0.08^{a}	0.56 ± 0.06^{b}	0.60 ± 0.01^{b}	0.83 ± 0.06^{a}
Ca	980 ± 90^{a}	590 ± 30^{b}	930 ± 30^{a}	810 ± 90^{a}	440 ± 60^{c}	627 ± 9^{b}	800 ± 60^{a}
Cd	0.154 ± 0.003^{a}	0.157 ± 0.004^{a}	0.138 ± 0.005^{b}	0.080 ± 0.002^{a}	0.081 ± 0.004^{a}	0.081 ± 0.006^{a}	0.083 ± 0.005^{a}
Cu	1.7 ± 0.2^{b}	3.3 ± 0.1^{a}	1.47 ± 0.07^{b}	4.6 ± 0.4^{bc}	5.68 ± 0.04^{ab}	6.7 ± 0.8^{a}	4.1 ± 0.1^{c}
Fe	Detected*	Detected*	Detected*	3.7 ± 0.4^{b}	2.6 ± 0.3^{c}	2.5 ± 0.3^{c}	4.9 ± 0.6^{a}
K	7000 ± 400^{c}	11400 ± 300^{a}	8800 ± 700^{b}	7200 ± 200^{c}	9000 ± 1000^{b}	7800 ± 400^{bc}	10800 ± 300^{a}
Mg	132 ± 5^{b}	209 ± 2^{a}	126 ± 8^{b}	200 ± 40^{a}	170 ± 10^{a}	160 ± 20^{a}	202 ± 4^{a}
Mn	1.42 ± 0.03^{b}	2.30 ± 0.04^{a}	1.7 ± 0.2^{b}	2.3 ± 0.4^{ab}	1.9 ± 0.3^{b}	2.0 ± 0.3^{b}	3.0 ± 0.4^{a}
Mo	0.131 ± 0.002^{b}	0.168 ± 0.009^{a}	0.136 ± 0.007^{b}	-	-	-	-
Na	-	-	-	Detected*	3.5 ± 0.3^{b}	3.4 ± 0.3^{b}	6.30 ± 0.01^{a}
Ni	Detected*	Detected*	Detected*	0.51 ± 0.03^{a}	0.48 ± 0.06^{a}	0.236 ± 0.006^{b}	0.32 ± 0.03^{b}
P	380 ± 10^{a}	310 ± 20^{b}	200 ± 20^{c}	420 ± 30^{b}	320 ± 10^{c}	348 ± 8^{bc}	700 ± 50^{a}
Sb	0.080 ± 0.001^{a}	0.084 ± 0.003^{a}	0.073 ± 0.003^{b}	-	-	-	-
Se	-	Detected*	-	-	-	-	-
Sn	Detected*	Detected*	Detected*	-	-	-	-
Ti	-	-	-	0.072 ± 0.008	Detected*	Detected*	0.075 ± 0.008
Zn	2.8 ± 0.1	Detected*	2.6 ± 0.3	5.54 ± 0.08^{a}	4.9 ± 0.1^{b}	3.22 ± 0.07^{d}	3.6 ± 0.2^{c}

*Concentrations between detection and quantitation limits.
Different superscripts (^{a}, ^{b} and ^{c}) indicate significant differences in the extracts (p<0.05).

Table S8. Average concentration levels of minerals and trace elements in the analyzed Picual olives in different months of two seasons; expressed in µg g^{-1} fresh weight. Results are given as mean ± standard deviation (n=3).

Picual	**2017/2018**			**2018/2019**			
**Element**	**2nd HD**	**3rd HD**	**4th HD**	**1st HD**	**2nd HD**	**3rd HD**	**4th HD**
Al	-	-	Detected*	-	Detected*	-	Detected*
As	0.26 ± 0.01^{a}	0.25 ± 0.01^{a}	0.23 ± 0.03^{a}	0.094 ± 0.005^{a}	0.10 ± 0.01^{a}	0.097 ± 0.002^{a}	0.091 ± 0.001^{a}
Ba	0.54 ± 0.01^{a}	0.35 ± 0.04^{b}	0.48 ± 0.01^{a}	0.099 ± 0.002^{b}	0.122 ± 0.004^{a}	0.106 ± 0.008^{ab}	0.10 ± 0.01^{b}
Ca	700 ± 80^{a}	340 ± 30^{b}	450 ± 40^{b}	277 ± 7^{b}	510 ± 40^{a}	300 ± 30^{b}	187 ± 9^{c}
Cd	0.160 ± 0.009^{a}	0.153 ± 0.006^{a}	0.15 ± 0.02^{a}	0.082 ± 0.004^{a}	0.083 ± 0.009^{a}	0.084 ± 0.001^{a}	0.079 ± 0.001^{a}
Cu	2.38 ± 0.09^{c}	2.776 ± 0.005^{b}	4.35 ± 0.07^{a}	3.5 ± 0.2^{b}	10 ± 1^{a}	4.0 ± 0.2^{b}	3.9 ± 0.4^{b}
Fe	-	Detected*	Detected*	Detected*	2.2 ± 0.3^{a}	1.84 ± 0.07^{a}	2.5 ± 0.4^{a}
K	5300 ± 100^{c}	6500 ± 500^{b}	10100 ± 400^{a}	6000 ± 200^{b}	7000 ± 500^{a}	5700 ± 220^{b}	6400 ± 400^{ab}
Mg	109 ± 2^{b}	110 ± 1^{b}	257 ± 4^{a}	150 ± 5^{a}	152 ± 5^{a}	119 ± 4^{b}	130 ± 10^{b}
Mn	1.46 ± 0.02^{b}	1.31 ± 0.03^{b}	2.0 ± 0.2^{a}	1.33 ± 0.04^{a}	1.4 ± 0.2^{a}	1.16 ± 0.04^{a}	1.4 ± 0.2^{a}
Mo	0.132 ± 0.007^{a}	0.15 ± 0.01^{a}	0.13 ± 0.01^{a}	-	-	-	-
Na	-	-	-	2.9 ± 0.1^{c}	4.05 ± 0.02^{a}	3.6 ± 0.2^{b}	4.05 ± 0.06^{a}
Ni	Detected*	Detected*	Detected*	0.18 ± 0.03^{a}	0.062 ± 0.006^{b}	0.093 ± 0.009^{b}	0.074 ± 0.009^{b}
P	260 ± 10^{b}	274 ± 3^{b}	458 ± 3^{a}	570 ± 40^{b}	550 ± 30^{b}	380 ± 20^{c}	830 ± 90^{a}
Sb	0.083 ± 0.005^{a}	0.082 ± 0.004^{a}	0.08 ± 0.01^{a}	-	-	-	-
Sn	Detected*	Detected*	Detected*	-	-	-	-
Ti	-	-	-	Detected*	Detected*	0.13 ± 0.03	Detected*
Zn	2.53 ± 0.04^{c}	4.3 ± 0.4^{a}	3.2 ± 0.2^{b}	2.53 ± 0.07^{a}	1.7 ± 0.1^{c}	2.45 ± 0.03^{ab}	2.3 ± 0.1^{b}

*Concentrations between detection and quantitation limits.
Different superscripts (^{a}, ^{b} and ^{c}) indicate significant differences in the extracts ($p<0.05$).

Figure S1. Recovery yields with the olives of the sample "Cornicabra 1st HD", spiked at 0.2, 10, and 20 µg g^{-1} fresh weight.

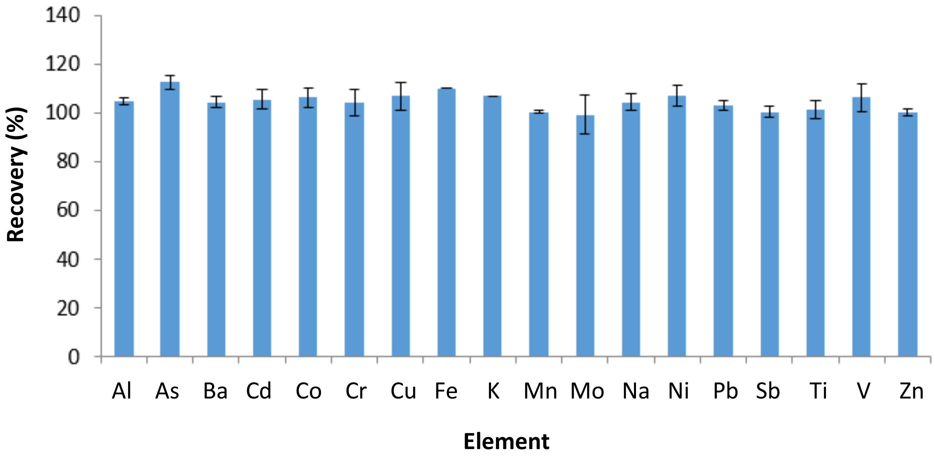

Supplement: Supplementary file 1 [file foods-10-00380-s001.pdf]
